# Rechallenge with anti-EGFR therapy to extend the continuum of care in patients with metastatic colorectal cancer

**DOI:** 10.3389/fonc.2022.946850

**Published:** 2023-02-02

**Authors:** Chiara Cremolini, Clara Montagut, Philippe Ronga, Filippo Venturini, Kensei Yamaguchi, Sebastian Stintzing, Alberto Sobrero

**Affiliations:** ^1^ Unit of Medical Oncology, Department of Translational Research and New Technologies in Medicine and Surgery, University of Pisa, Pisa, Italy; ^2^ Department of Medical Oncology, Hospital del Mar— Institut Hospital del Mar d'Investigacions Mèdiques (IMIM), Centro de Investigación Biomédica en Red de Cáncer (CIBERONC), Barcelona, Spain; ^3^ Global Medical Affairs, Merck Healthcare KGaA, Darmstadt, Germany; ^4^ Global Medical Affairs, Merck Serono S.p.A., an Affiliate of Merck KGaA, Rome, Italy; ^5^ Department of Gastroenterological Chemotherapy, Cancer Institute Hospital of Japanese Foundation for Cancer Research, Tokyo, Japan; ^6^ Department of Hematology, Oncology, and Cancer Immunology (CCM), Charité–Universitätsmedizin, Berlin, Germany; ^7^ Department of Medical Oncology, Ospedale San Martino, Genoa, Italy

**Keywords:** metastatic colorectal cancer (mCRC), anti-EGFR, rechallenge, reintroduction, liquid biopsy

## Abstract

In patients with *RAS* wild-type metastatic colorectal cancer (mCRC), an anti-epidermal growth factor receptor (EGFR) monoclonal antibody plus chemotherapy is a standard option for treatment in the first-line setting. Patients who progress while on treatment with anti-EGFR-based therapy can be resistant to further anti-EGFR treatment, but evidence suggests that the anti-EGFR-resistant clones decay, thereby opening the potential for rechallenge or reintroduction in later lines of treatment. Results from recent clinical studies have shown that some patients with mCRC who are rechallenged with anti-EGFR monoclonal antibodies exhibit durable responses. While other therapies have demonstrated improved overall survival in chemorefractory mCRC over the past decade, rechallenge with anti-EGFR monoclonal antibodies in later lines of treatment represents a new option that deserves further investigation in clinical trials. In this review, we summarize the molecular rationale for rechallenge or reintroduction in patients with mCRC who have progressed on earlier-line anti-EGFR treatment and examine the current evidence for using liquid biopsy as a method for selecting rechallenge as a therapeutic option. We also provide an overview of published trials and trials in progress in this field, and outline the potential role of rechallenge in the current clinical setting.

## 1 Introduction

Colorectal cancer (CRC) is the second leading cause of cancer mortality worldwide, with 935,173 deaths and 1,931,590 new cases in 2020 (1). Approximately 40% of patients with CRC eventually develop metastatic disease (2). Median overall survival (OS) has improved in patients with metastatic CRC (mCRC) over the past two decades, as a result of increased screening, better supportive care, and the development of new and more optimized palliative systemic treatments such as targeted therapy and improved chemotherapy regimens (3). However, third- and fourth-line treatment of mCRC remains an unmet clinical need and expansion of the continuum of care is needed for continued progress in providing clinical benefit (4–7).

Several targeted agents that are recommended for the first-line treatment of mCRC in combination with chemotherapy are currently available (8, 9). These agents include the anti–epidermal growth factor receptor (EGFR) monoclonal antibodies (mAbs) cetuximab and panitumumab for patients bearing *RAS* wild-type (wt) tumors, as well as the anti-vascular endothelial growth factor (anti-VEGF) mAb bevacizumab (2, 10). Despite the successes of targeted therapy in improving the lives of patients with mCRC, the development of acquired resistance still represents a major challenge in the use of these targeted agents (7). Point mutations in the antibody-binding site in the extracellular domain (ECD) of *EGFR* (*EGFR*-ECD) can arise over time due to the selective pressure of anti-EGFR treatment (11–13), and constitutive activation of downstream signaling via *KRAS*, *NRAS*, and other mutations can also confer resistance (14–16). Such mutations convert initially sensitive tumors into resistant tumors, thus discouraging the further use of anti-EGFR mAbs if resistance is detected.

Following their emergence in tumors that have become resistant to anti-EGFRs, mutant (mt) clones of *RAS* and *EGFR*-ECD begin to decay after anti-*EGFR* withdrawal, with half-lives of 3.4 and 6.9 months, respectively, and a cumulative half-life of 4.4 months (17). This putative decay of *RAS* and *EGFR-*ECD mt clones provides a rationale for the possibility of rechallenging patients who received anti-EGFR therapy in an earlier line of treatment, developed resistance, and received an intervening line of treatment free of anti-EGFR therapy (7, 18).

Different clinical scenarios can be considered based on the time interval between the last administration of the anti-EGFR agent in first-line treatment and the evidence of progressive disease (PD). True rechallenge occurs when a patient who is initially responding to anti-EGFR-based therapy (clinical benefit of complete response [CR], partial response [PR], or stable disease [SD], ideally for ≥6 months) develops PD while on this treatment, switches to a non-anti-EGFR regimen until PD, and is then rechallenged with anti-EGFR-based therapy (7, 19). Studies examining true rechallenge with anti-EGFR therapy, such as CRICKET, E-Rechallenge, and CHRONOS have demonstrated that patients whose disease progressed on a first-line anti-EGFR–based regimen may achieve benefit from rechallenge with anti-EGFR therapy in the third line or later lines (20–23). Related terms such as reintroduction and retreatment may be confused with rechallenge, but there are differences.

Reintroduction occurs when a patient who is responding to an anti-EGFR-based regimen stops treatment for a reason other than PD (e.g., due to toxicity or preference) and is subsequently restarted on anti-EGFR-based therapy at a later time. This is not considered to be a rechallenge because the tumor did not become resistant to treatment during therapy (7). Retreatment is a more general term that means anti-EGFR-based therapy is being administered again; both rechallenge and reintroduction fall under the category of retreatment. It should be noted that the terms rechallenge, reintroduction, and retreatment are sometimes used interchangeably; it is important to know the details of the trial design to confirm which type of treatment modality is being examined.

In this review, we summarize the molecular rationale for rechallenge or reintroduction in patients with mCRC who have progressed on earlier-line anti-EGFR treatment and examine the current evidence for using liquid biopsy as a method for selecting rechallenge as a therapeutic option. In addition, we provide an overview of published and ongoing trials in this field and outline the potential role of rechallenge in the current clinical setting.

## 2 Translational evidence for rechallenge and liquid biopsy

Mutations that confer resistance to anti-EGFR therapy can arise during treatment (24). Mutations in exons 2, 3, and 4 of the *KRAS* gene as well as mutations in exons 2, 3, and 4 of the *NRAS* gene (together considered *RAS* mutations) are negative predictive biomarkers for anti-EGFR-targeted therapy (25–28). Analyzing circulating tumor DNA (ctDNA) has revealed mechanisms of primary or acquired resistance to EGFR blockade, such as alterations in *KRAS*, *NRAS*, *HER2*, *MET*, *ERBB2*, *EGFR*-ECD, *BRAF*, and *MAP2K1* (29, 30). These same molecular alterations may be present at the start of treatment in low allelic frequencies in tumors classified as *RAS* wt according to techniques commonly adopted in daily clinical practice, with the exception of *EGFR*-ECD mutations (which have never been detected before anti-EGFR therapy) (31, 32).

Acquired mutations in the *EGFR*-ECD can block the binding of anti-EGFR mAbs and lead to resistance (11, 12, 24, 30, 32). In the study by Montagut et al, in 193 patients with mCRC treated with anti-EGFR therapy, liquid biopsy revealed *RAS* mutations in 29.5% of cases and *EGFR*-ECD mutations in 25% of cases as the most frequent mechanisms of acquired resistance (30). In a comprehensive analysis of ctDNA in mCRC patients, patients with *EGFR*-ECD mutations displayed striking tumor heterogeneity, with 91% harboring distinct multiple mechanisms of resistance (33). Thus, a tumor can develop multiple mechanisms of resistance at once, which are mostly subclonal; this heterogeneity of resistant alterations calls for new treatment strategies to overcome anti-EGFR resistance (30, 33–35).

Levels of *RAS* mt clones can change over time, thus making rechallenge with anti-EGFR mAbs a viable option in later lines of treatment. *RAS* mt clones display pulsatile behavior, arising with EGFR blockade and decaying on withdrawal of the anti-EGFR mAb, allowing a tumor to regain sensitivity to the drug (29). *RAS* and *EGFR* mt clones decay exponentially with a cumulative half-life of 4.4 months after discontinuation of anti-EGFR therapy, with retrospective analyses leading to the hypothesis that objective response rates (ORRs) are higher when patients are rechallenged after increasing time intervals following previous treatment with anti-EGFR mAbs (17, 36). Indeed, response to an intervening line of non-anti-EGFR therapy (initiated after resistance to initial anti-EGFR treatment has developed) may provide an opportunity for *RAS* wt clones to reemerge, thus allowing anti-EGFR sensitivity to be regained. Although mutant clone decay has never been proven in tissue samples, liquid biopsies have provided evidence of this mechanism (16).

Liquid biopsies may be used to analyze ctDNA and provide information about a patient’s eligibility for any line of treatment with anti-EGFR mAbs and subsequent rechallenge with this therapy, as the analysis of ctDNA found in the bloodstream can reveal genetic changes occurring in the tumor (37). Traditional tissue biopsies or excisional biopsies on the tumor itself can be invasive or risky, and these are not able to properly depict intra- and inter-metastatic heterogeneity (8, 38). More than 75% of patients with advanced CRC have detectable ctDNA (39, 40), and highly sensitive techniques are required for ctDNA analysis. Methods used for ctDNA detection include polymerase chain reaction-based approaches—such as BEAMING (beads, emulsion, amplification and magnetics), droplet digital polymerase chain reaction, and Idylla™—or massively parallel deep sequencing or next-generation sequencing approaches using molecular barcoding and appropriate bioinformatics analysis to improve sensitivity and reduce artifacts (41–43). Current liquid biopsy techniques and assays have both advantages and limitations, with regards to their clinical utility. While single-gene panels are cost-effective, they may not detect variants in the genes that are not being evaluated but could also influence therapy (44). On the other hand, multi-gene panels are associated with higher cost and decreased depth of gene sequencing but can better assess prognosis and detect recurrence (44). The 2022 European Society for Medical Oncology (ESMO) recommendations specifically state that validated and sensitive liquid biopsy assays may be used clinically to test *KRAS/NRAS/BRAF/EGFR*-ECD mutations in pretreated patients with mCRC, if EGFR rechallenge is planned, keeping in mind the limitation of incomplete insensitivity of such tests (45). A recent study has confirmed that liquid biopsy can be utilized to select *RAS/BRAF* wt mCRC patients at rechallenge baseline, though the sample size for this retrospective analysis was quite small. This study also suggested that combining results from ctDNA analysis with clinical predictive factors (such as previous response to anti-EGFR therapy and anti-EGFR-free interval) may be effective (46). It is important to note that clinically relevant threshold levels for *RAS* mt as determinants of resistance to anti-EGFR therapy are not established, and other markers may need to be considered as well, specifically *EGFR*-ECD mutations (which are present in 25% of resistant tumors) and less frequent markers such as *BRAF, HER2*, *MET*, *MAP2K1*, and others. A combination of liquid biopsies with mathematical modelling of tumor evolution may enable the design of optimal personalized treatments in the future (30).

## 3 Clinical evidence for rechallenge with anti-EGFR therapy

Several studies have been published in recent years detailing the use of rechallenge in patients with *RAS* wt mCRC, suggesting that this treatment strategy may be clinically beneficial (**Table 1**). Patients who are rechallenged with anti-EGFR therapy receive the targeted treatment either with or without concomitant chemotherapy in a later line and usually after ≥3 months of an intervening anti-EGFR-free therapy. This treatment generally demonstrates an acceptable safety profile and no unexpected toxicities; the incidence of grade 3/4 adverse events (AEs) is generally low, with no treatment-related deaths reported and infrequent discontinuations due to toxicity.

**Table 1:** Summary of rechallenge/reintroduction trials with anti-EGFR agents.

Study	Design	N	Patient population	Site (n)	Line	RR, %	SD, %	DCR, %	PFS, months	OS, months	Common grade 3/4 AEs, %
Santini et al. 2012 (19)	Phase II	39	*KRAS* codons 12–13 wt	Colon (19); rectum/rectosigmoid (20)	≥4	53.8	35.9	89.8	6.6	NR	Skin rash (38.5), neutropenia (18.0), diarrhea (7.7)
JACCRO CC-08 (47)	Phase II	34	*KRAS* wt	Right colon (4), left colorectum (30)	3	2.9	53.0	55.9	2.4	8.2	Neutropenia (28.6)
JACCRO CC-09 (48)	Phase II	25	*KRAS* wt exon 2	NR	3	8.3	41.7	50	3.1	8.9	Acneiform rash (17), hypomagnesemia (13) and dry skin (13)
Nogueira et al., 2016 (49)	Retrospective	15	*KRAS* wt	Colon (10); rectum (5)	≥3	13.3*	26.7	40.0	3.5	36.9	Skin toxicity (13.3)
Tanioka et al., 2018 (50)	Retrospective	14	*KRAS* wt exon 2	NR	3–6	21.4	50.0	71.4	4.4	NR	Rash (7), diarrhea (7)
E-Rechallenge (21)	Phase II	33	*RAS* wt	Right colon (4); left colon (29)	NS	15.6*	40.6	56.2	2.9	8.7	NR
CRICKET (20)	Phase II	28	*RAS*/*BRAF* wt	Right colon (9); left colon or rectum (19)	3	21	31	54	*RAS* wt: 4.0	*RAS* wt: 12.5	Diarrhea (18), acneiform skin rash (14), neutropenia (14), hand and foot syndrome (7)
E-Rechallenge (additional study) (22)	Phase II	24	*RAS* wt	NR	NS	12.5*	50.0	62.5	NR	NR	NR
TRECC (51)	Retrospective	68	*KRAS/NRAS* wt	Right/ transverse colon (4); left/rectum (64)	≥3	18	NR	NR	3.3	7.5	NR
CAVE (52)	Phase II	77	*RAS* wt	NR	3	7.8	57.1	65	3.6	11.6	Cutaneous eruption (14), diarrhea (4)
CHRONOS (23)	Phase II	27	*RAS*/*BRAF* wt	Right colon (5); left colon (17); rectum (5)	≥4	30*	40	63	16 weeks	55 weeks	Dermatological (22)
PURSUIT (53)	Phase II	50	*RAS*/*BRAF* wt	Left-sided primary (44)	≥2	14	66	80	3.6	NR	NR

In the TRECC study, only 25% of patients were rechallenged with an anti-EGFR agent and 75% were reintroduced to an anti-EGFR agent. *Partial response.

AE, adverse event; DCR, disease control rate; EGFR, epidermal growth factor receptor; NR, not reported; NS, not specified; OS, overall survival; PFS, progression-free survival; RR, relative risk; SD, stable disease; wt, wild-type.

A prospective study collected data on 39 patients with *KRAS* wt mCRC (in codons 12 and 13), who had shown clinical benefit with cetuximab plus irinotecan (or FOLFIRI) in a prior line, developed disease progression, received intervening treatment with chemotherapy alone, and were rechallenged with cetuximab plus irinotecan-based therapy thereafter (19). The median time interval between the last cycle of initial cetuximab therapy and the first cycle of cetuximab rechallenge was 6 months (range: 2–12 months). The median number of therapy lines before cetuximab rechallenge was 4 lines (range: 3–7 lines) (19). The ORR after rechallenge was 53.8%, with PRs in 19 patients (48.7%) and CRs in 2 patients (5.1%). A total of 35.9% of patients had SD, while 4 patients (10.2%) progressed; median progression-free survival (PFS) after rechallenge was 6.6 months. A total of 94.9% of patients developed skin rash as a result of treatment; the most frequent grade 3/4 AEs were skin rash and diarrhea. Two patients (5%) discontinued treatment due to toxicity, and no deaths occurred (19). This was the first demonstration that rechallenging patients with cetuximab could have clinical benefit.

Following the signals of benefit reported by the above-referenced study by Santini et al. (19), the phase II JACCRO CC-08 study was initiated in Japan to investigate rechallenge with cetuximab (47). Thirty-four patients with *KRAS* wt mCRC were rechallenged with cetuximab plus irinotecan after showing clinical benefit (SD for ≥6 months or response) and subsequent progression in response to first-line cetuximab plus chemotherapy as well as progression after second-line chemotherapy. The median cetuximab-free interval was 330 days (range: 56–1224 days). The ORR was 2.9% with a disease control rate (DCR) of 55.9%, the median PFS was 2.4 months, and the median OS was 8.2 months. In the JACCRO CC-08 study, neutropenia was the most frequent grade 3/4 AE, occurring in 28.6% of patients; the most common grade 1/2 skin toxicity (skin rash) occurred in 80% of all patients (47). A *post hoc* biomarker study examined the association between *RAS* status and clinical outcomes in the JACCRO trials. A total of 16 patients (4 from JACCRO-CC 08 and 12 from JACCRO-CC 09) with known *RAS* status by ctDNA at baseline, 8 weeks, and progression after initial therapy were enrolled. This *post hoc* analysis demonstrated that *RAS* status in ctDNA predicts survival of rechallenge treatment with an anti-EGFR agent. Patients with *RAS* mutations in ctDNA at baseline had a significantly shorter PFS and significantly poorer OS than those without mutations (median PFS, 2.3 vs 4.7 months, respectively; hazard ratio [HR], 6.2; *P*=0.013; and median OS, 3.8 vs 16.0 months, respectively; HR, 12.4; *P*=0.0028) (54).

A retrospective study in Portugal examined 15 patients with *KRAS* wt mCRC who progressed after initial cetuximab plus irinotecan treatment and progressed again after subsequent chemotherapy before receiving cetuximab plus irinotecan as a rechallenge therapy (49). The median anti-EGFR-free interval (time between last cycle of first line of cetuximab and first cycle of cetuximab retreatment) was 7.7 months and the median number of treatment lines prior to rechallenge was 4 (range: 2–6). No information was provided regarding the specific treatment regimens used. Along with cetuximab, 73.3% of patients received irinotecan and 26.7% received FOLFIRI for rechallenge. In total, 13.3% of patients experienced a PR, 26.7% of patients had SD, and 46.7% of patients progressed; the remainder were non-evaluable or their status was unknown. The median PFS was 3.5 months and the median OS was 36.9 months. During rechallenge, 86.7% of patients developed grade 1/2 skin toxicities and 2 (13.3%) cases of grade 3 skin toxicities were reported.

Another retrospective study in Japan analyzed 14 patients with *KRAS* wt exon 2 mCRC who initially received cetuximab as a first-line (11 patients), second-line (1 patient), or third-line treatment (2 patients) (50). During initial treatment with cetuximab, 12 patients had a PR and 2 patients had SD lasting ≥6 months. The median time interval between initial cetuximab and rechallenge was 12.6 months (range: 6.6–37.1 months); rechallenge occurred as third-, fourth-, fifth-, and sixth-line treatments in 6, 1, 2, and 5 patients, respectively. Intervening therapies comprised chemotherapy in combination with bevacizumab, TAS-102, and regorafenib. All patients received cetuximab combined with irinotecan as the rechallenge therapy. The ORR after rechallenge was 21.4% (all PRs in 3 patients); the median PFS was 4.4 months. Seven patients had SD, leading to an overall DCR of 71%. In terms of AEs, all patients developed skin rash, including 1 grade 3/4 toxicity; there was also 1 case of grade 3/4 diarrhea. No patients discontinued due to AEs, and no infusion reactions associated with cetuximab were reported. This study showed that patients with an initial response to cetuximab may be good candidates for rechallenge with cetuximab and that long intervals between administrations of cetuximab—even with multiple intervening treatment lines—are important for deriving clinical benefit from rechallenge.

The above-referenced study reported by Tanioka et al. (50) validates the potential for rechallenging patients with cetuximab beyond third-line therapy. Patients with *RAS* wt mCRC can also be administered cetuximab as a second-line therapy (55, 56). Current recommendations also suggest that cetuximab can be used as third- or later-line therapy in patients who have received chemotherapy, bevacizumab, and/or regorafenib in earlier lines (57). However, anti-EGFR therapy may not always be effective after certain treatment strategies; in particular, studies show conflicting results when administering cetuximab after first-line treatment with bevacizumab (57–59).

The CRICKET study was a prospective phase II trial that examined rechallenge with cetuximab and irinotecan in patients with *RAS* and *BRAF* wt mCRC who acquired resistance to first-line cetuximab and FOLFIRI or FOLFOXIRI before undergoing second-line treatment with bevacizumab and FOLFOX, FOLFOXIRI, or XELOX (20). Of note, patients were eligible for the CRICKET study if ≤4 weeks had elapsed between the last administration of anti-EGFR therapy in first line and disease progression being confirmed after first-line therapy. Moreover, they were required to have experienced at least a PR with first-line cetuximab and FOLFIRI or FOLFOXIRI and a PFS of ≥6 months with first-line therapy. Liquid biopsy was used retrospectively to verify *RAS* and *BRAF* status at the start of rechallenge. In the overall population (not selected by liquid biopsy), 6 PRs and 9 cases of SD were reported among 28 patients enrolled, thereby resulting in an ORR of 21%; the DCR was 54%, the median PFS was 3.4 months, and the median OS was 9.8 months. These data are comparable to the outcomes of a population of *EGFR*-positive patients unselected for *RAS* status and treated with cetuximab and irinotecan (60). In the CRICKET study (20), patients with *RAS* wt ctDNA had significantly longer PFS (4.0 months) compared with patients with *RAS* mt ctDNA at the start of rechallenge therapy (1.9 months; *P*=0.03), although no significant differences in median OS were observed. Furthermore, of 25 patients evaluable for a radiological response, 13 showed tumor shrinkage. Only patients who were ctDNA *RAS* and *BRAF* wt benefited from rechallenge in terms of response; no *RAS* mutations were detected in patients who achieved a confirmed PR, while 12 of 21 patients who did not achieve a response had *RAS* mutations detectable in ctDNA before starting retreatment. These results support the use of liquid biopsy before rechallenge, as well as the potential need to investigate other markers of resistance beyond *RAS* (such as *EGFR*-ECD or *BRAF*) to more accurately select patients who may benefit from cetuximab rechallenge. One patient with a low level of *RAS* mutation in ctDNA experienced a transient response to rechallenge, highlighting the validity of using mutational status as a threshold rather than as a yes/no indicator.

CRICKET was the first published prospective study showing that rechallenge is feasible and has clinical benefit in patients with *RAS* and *BRAF* wt mCRC after acquiring resistance to first-line cetuximab plus irinotecan-based chemotherapy (20). The data also support the use of liquid biopsy to assess the potential benefit of rechallenge in patients who have progressed on earlier treatment with cetuximab plus chemotherapy. However, because not all patients with *RAS* wt tumors respond to rechallenge, other mechanisms of resistance are also important in determining response and should be taken into consideration when performing liquid biopsies. Although prognostic and potentially predictive markers such as *BRAF* and *PIK3CA* mutations were not identified from analyzed samples in the CRICKET study, other mutations such as those in *EGFR*-ECD may also affect clinical efficacy (20).

The E-Rechallenge trial in Japan (**Table 1**), a multicenter phase II study, examined rechallenge in patients with mCRC refractory to fluoropyrimidines, oxaliplatin, irinotecan, cetuximab, and bevacizumab, and who have responded to cetuximab with SD for ≥6 months, PR, or CR in any earlier line (21, 22). Thirty-three patients were enrolled and, in preliminary results, 15.6% of patients achieved a PR, 40.6% of patients had SD, and 43.8% of patients had PD during rechallenge. Twenty-four of 33 patients participated in additional retrospective liquid biopsy screenings of ctDNA; in patients with *RAS*, *BRAF*, and *EGFR* wt variants of these genes, the PR rate increased to 25%, and 50% of these patients had SD, resulting in a DCR of 75%. These data show that screening for wt status in *RAS*, *BRAF*, and *EGFR*-ECD genes may aid in predicting the effectiveness of rechallenge. Although CRICKET and E-Rechallenge support the ideas that sensitivity to cetuximab can be regained after the development of resistance and that liquid biopsy is a feasible method to determine eligibility, the potential for efficacy should be the main driving factor for rechallenge (21, 22).

In a retrospective study of patients who received first-line panitumumab plus FOLFOX, the median OS was 14.2 months after initiation of rechallenge with panitumumab in the third or later line, while a phase II study of Japanese patients who received panitumumab-based therapy as both first-line therapy and third-line rechallenge had a median PFS of 3.8 months, median OS of 8.9 months, and ORR of 8.3% (61, 62). The CHRONOS trial (NCT03227926; a single-arm phase II trial in Italy), enrolled 27 patients with *RAS/BRAF/EGFR* wt who had achieved an objective response followed by progression in any treatment line (median number of previous treatment lines was 3) with an anti-EGFR mAb regimen (23). The primary endpoint of ORR after treatment with panitumumab was 30%, with PRs in 8 patients (30%) and SD in 11 patients (40%); the median PFS was 16 weeks (23).

The TRECC study in Brazil was a retrospective study that investigated 68 patients who received an anti-EGFR mAb (cetuximab or panitumumab) plus chemotherapy after discontinuing the treatment in an earlier line; the median length of the anti-EGFR-free interval was 10.5 months (51). It should be noted that only 25% of the patient population had discontinued anti-EGFR-based therapy due to PD during the first usage. Although these patients fall under the category of true rechallenge, the regimen for the other 75% of patients who discontinued for reasons other than PD would be considered as reintroduction. Furthermore, in the TRECC study, 59 patients received cetuximab and 9 patients received panitumumab at retreatment. The median PFS after retreatment was 6.6 months, while the median OS was 24.4 months, and a multivariate analysis determined that PD as a reason for first discontinuation was the only adverse prognostic factor related to PFS (51).

The phase II, single-arm CAVE-Colon trial in Italy enrolled 77 patients with *RAS* wt mCRC with the intent of rechallenging them with cetuximab plus avelumab in the third line after first-line use of anti-EGFR mAb plus chemotherapy (only for patients characterized by CR/PR/SD) and after subsequent second-line treatment (52), which in clinical practice most commonly includes an anti-VEGF agent plus chemotherapy, as recommended by the NCCN Clinical Practice Guidelines in Oncology (NCCN Guidelines^®^) (9). In this trial, the combination of cetuximab plus avelumab resulted in a median OS of 11.6 months (95% CI, 8.4–14.8 months) and median PFS of 3.6 months (95% CI, 3.2–4.1 months). Based on the OS benefit observed in the CAVE trial, this chemotherapy-free rechallenge strategy may be an effective new treatment option for patients with *RAS* wt mCRC who have progressed after first-line anti-EGFR-based chemotherapy (52). A recently published *post hoc* analysis of this trial investigated the role of skin toxicity as a biomarker of clinical response in the rechallenge setting and found a correlation between high-grade skin toxicity and survival. Patients who had experienced grade 2/3 skin toxicity had significantly longer median OS (17.8 months vs 8.2 months; HR, 0.51; *P*=0.019) and median PFS (4.6 months vs 3.4 months; HR, 0.49; *P*=0.004), compared with patients who experienced grade 0/1 skin toxicity (63). In the exploratory CAVE trial analysis, which investigated the predictive role of neutrophil-to-lymphocyte ratio (NLR), lower NLR was significantly correlated with improved OS (64). Median OS was significantly longer in patients (intention-to-treat population) with NLR <3 compared with those with NLR ≥3 (17.8 months vs 8.9 months; HR, 0.50; 95% CI, 0.3–0.8; *P*=0.006). In the ctDNA *RAS/BRAF* wt population, patients with NLR <3 had a significant improvement in median OS compared with those with NLR ≥3 (22.0 months vs 8.9 months; HR, 0.38; 95% CI, 0.19–0.75; *P*=0.005). These results suggest that NLR could be a potential biomarker of response to the combination of cetuximab plus avelumab (64).

In the JACCRO CC-09 trial (a single-arm phase II trial in Japan), in 25 patients with *KRAS* wt mCRC, the rechallenge with panitumumab plus irinotecan resulted in a response rate of 8.3%; median PFS and median OS were 3.1 months and 8.9 months, respectively (48). Several clinical trials investigating panitumumab rechallenge or reintroduction are ongoing (**Table 2**), including VELO (EudraCT Number 2018-001600-12) (65). The VELO trial, a randomized, multicenter phase II trial, is comparing panitumumab plus TAS-102 (trifluridine/tipiracil) vs TAS-102 as third-line treatment in 112 patients with *RAS* wt mCRC, who had achieved PR or CR in first-line treatment with anti-EGFR therapy; the primary endpoint is PFS, while secondary endpoints include OS and ORR (65, 71).

**Table 2:** Summary of ongoing rechallenge/reintroduction trials with anti-EGFR agents.

Study	Phase	N	Patient population	Therapy	Line	Primary endpoint	Secondary endpoint(s)	Registration number
VELO (65)	Phase II	112	*RAS* wt	Panitumumab + trifluridine/tipiracil vs. trifluridine/tipiracil	3	PFS	OS, ORR	EudraCT Number 2018-001600-12
FIRE-4 (AIO KRK-0114) (66)	Phase III	230	*RAS* wt	Cetuximab + FOLFIRI vs. regorafenib	3	OS	RR, PFS, safety, DpR, early tumor shrinkage, biomarker analysis	NCT02934529
CITRIC (67)	Phase II	66	*RAS, BRAF, EGFR-*ECD wt	Cetuximab + irinotecan	3	ORR	DCR, DOR, PFS, OS, safety	EudraCT Number 2020-000443-31
PARERE (68)	Phase II	214	*RAS* and *BRAF* wt	Panitumumab > regorafenib vs. regorafenib > panitumumab	3	OS	PFS (1^st^ and 2^nd^), TTF, ORR, safety	EudraCT Number 2019-002834-35/NCT04787341
PULSE (69)	Phase II	120	*RAS* wt	Panitumumab vs. regorafenib or trifluridine/tipiracil	NS	OS	PFS, ORR, CBR, safety, QoL	NCT03992456
CAPRI-2 GOIM (69)	Phase II	200	*RAS/BRAF* wt	Irinotecan + cetuximab*	≥2	RR	PFS, OS, safety, QoL	NCT05312398
CAVE-2 GOIM (70)	Phase II	173	*RAS/BRAF* wt	Avelumab and cetuximab vs. cetuximab alone	3	OS	ORR, PFS, safety	NCT05291156
MD Anderson Cancer Center study (USA) (65)	Phase II	84	*RAS, BRAF, EGFR-*ECD wt	Panitumumab	3	ORR	CR/PR/SD, PFS, OS	NCT03087071 (cohort 3)

These trials are ongoing at the time of manuscript submission. *RAS mt patients received trifluridine/tipiracil or regorafenib.

CBR, Clinical benefit rate; CR, complete remission; DCR, disease control rate; DOR, duration of response; DpR, depth of response; ECD, extra-cellular domain; EGFR, epidermal growth factor receptor; mt, mutant; NS, not specified; ORR, overall response rate; OS, overall survival; PD, progressive disease; PFS, progression-free survival; PR, partial remission; QoL, quality of life; RR, response rate; SD, stable disease; TTF, time to failure; wt, wild-type.

## 4 Clinical perspectives

A variety of clinical studies have shown the potential efficacy of using anti-EGFR-based therapy to rechallenge patients with mCRC after receiving an earlier anti-EGFR treatment regimen. Despite the feasibility of using liquid biopsy to determine *RAS* mutational status and identify patients who may benefit from rechallenge with anti-EGFR therapy, the technique is still not widely available internationally and is often used only in conjunction with research trials, so that the reliability of clinical factors in identifying patients who may benefit from rechallenge is investigated. Based on the published literature, a minimum of 6 months is frequently set as the time interval required between anti-EGFR treatment regimens; furthermore, prior responders who have a greater duration between anti-EGFR treatment intervals seem more likely to respond to rechallenge (36). However, recently published data from a multi-institutional analysis of 86 patients retreated with anti-EGFR therapy suggest that clinical factors such as the number of anti-EGFR-free lines of therapy, the length of the anti-EGFR-free time interval, the primary tumor side, and the time from diagnosis to retreatment are not associated with rechallenge response rate or PFS (72).

Rechallenge is a valuable treatment strategy due to its efficacy and the known safety profile of cetuximab plus irinotecan in patients who have already received the drug in an earlier line. Other later-line therapies, such as third-line trifluridine/tipiracil (ORR, 1.6%; DCR, 44%; OS, 7.1 months; grade ≥3 AEs, 69%) and second- or later-line regorafenib (ORR, 1.0%; DCR, 41%; OS, 6.4 months; grade ≥3 AEs, 54%), are also available (66, 73–75). Rechallenge with an anti-EGFR mAb has demonstrated encouraging clinical activity along with a tolerable safety profile, and liquid biopsy can be useful in the selection of patients eligible for rechallenge treatment.

Many patients still exhibit good disease control after chemotherapy and good long-term performance status while remaining suitable for additional therapy after progression beyond third- or fourth-line treatment; rechallenge with an anti-EGFR mAb may expand the continuum of care for these patients and may be considered multiple times in the therapeutic route of patients with EGFR-dependent tumors.

## 5 Future perspectives

Rechallenge using anti-EGFR mAbs is one of the most dynamic settings in stage IV CRC management: this review summarizes the state-of-the-art evidence through a picture of latest data which could be useful for clinicians. While there is evidence that rechallenge with anti-EGFR therapy may be effective in mCRC, defining the subset of patients that will benefit from rechallenge is still an unmet clinical need. Studies have investigated whether a combination of genotyping and clinical predictive factors (such as long anti-EGFR free interval and previous response to anti-EGFR therapy) can help define those patients who may benefit from the rechallenge strategy (46) or if only liquid biopsy-based genetic profiling [e.g., for plasma *RAS* mutations (53)] is sufficient for patient selection. Although the CRICKET study was the first to establish the use of liquid biopsy as a means of measuring *RAS* status as a mechanism of resistance to anti-EGFR treatment, several outstanding questions still exist concerning this approach. *RAS* mt levels differ in patients, and more work needs to be done regarding recommended threshold values for these levels to identify patients eligible for rechallenge. Moreover, some patients who were originally *RAS* wt continue to exhibit disease control even after *RAS* mt clones reemerge after rechallenge (8). Furthermore, high ratios of cells with *RAS* mt clones may reduce the efficacy of anti-EGFR mAbs. While liquid biopsy can determine eligibility for rechallenge, other markers and clinical characteristics can also be examined prior to initiation of rechallenge. Additional studies are needed to determine the optimal time for rechallenge with anti-EGFR therapy. Liquid biopsy has thus far been used retrospectively to assay ctDNA for mutational status. Current studies (**Table 2**) using liquid biopsy include the randomized FIRE-4 (AIO KRK-0114) trial (which is comparing cetuximab rechallenge with other treatment regimens in the third line) (66), the CITRIC trial (a randomized trial comparing cetuximab rechallenge to investigator’s choice in liquid biopsy triple negative *RAS*, *BRAF*, *EGFR*-ECD wt tumors) (67), the PARERE study (NCT04787341) comparing panitumumab rechallenge followed by regorafenib vs the reverse sequence in patients with *RAS* and *BRAF* wt ctDNA (68) and the PULSE trial (NCT03992456) comparing rechallenge with panitumumab vs regorafenib or trifluridine/tipiracil in refractory *RAS* wt mCRC (69). Future studies should include prospective analyses that better characterize the use of liquid biopsy to track and identify resistance.

Additional studies are underway to further evaluate the efficacy and safety of rechallenge with cetuximab and panitumumab. The phase III FIRE-4 (AIO KRK-0114; NCT02934529) trial in Germany is the first trial to study cetuximab rechallenge with randomization between third-line rechallenge with cetuximab plus irinotecan or FOLFIRI or physician’s choice; this trial includes the largest number of patients (N=450 in the first line, with 230 planned to receive rechallenge therapy) investigated for cetuximab rechallenge thus far (66). In Italy, a large, multicenter, phase II study (CAPRI-2) in 200 mCRC patients with *RAS/BRAF* wt tumors is examining two sequence treatment strategies to determine the optimal anti-EGFR therapy based on cancer molecular evolution (69). Cetuximab in third-line treatment is currently being investigated in patients with *RAS* wt mCRC who had a PR/CR with first-line anti-EGFR therapy and subsequently received a second line of treatment (65). Furthermore, a phase II trial (CAVE-2 GOIM study) investigating whether the chemotherapy-free rechallenge approach with the combination of avelumab and cetuximab offers a clinical advantage over cetuximab alone in patients with pretreated *RAS/BRAF* wt mCRC is currently underway (NCT05291156) (70). Ongoing trials are also evaluating panitumumab rechallenge and reintroduction (71).

## 6 Discussion

The continuum of care is ever evolving in the treatment of patients with mCRC. The results of the recently completed FRESCO-2 trial with fruquintinib in refractory mCRC show a significant and clinically meaningful improvement in OS (76). Regorafenib and trifluridine/tipiracil are other current treatment options beyond the second line that have been shown to improve OS in patients previously treated with chemotherapy or targeted therapy (5). However, high-quality evidence to support recommendations for the treatment of mCRC beyond the second line is limited (5) and new strategies are needed to maximize options for patients who have progressed on prior lines of therapy. For patients who initially responded to anti-EGFR therapy prior to acquiring resistance, rechallenge with an anti-EGFR agent has demonstrated activity. Optimal scenarios for administering anti-EGFR therapy in the rechallenge setting may include prior responsiveness to this treatment in patients with *RAS* wt mCRC and an appropriate (≥6 months) interval between anti-EGFR-based treatment regimens; in the rechallenge setting, the use of an anti-EGFR agent is not limited to a specific line. However, as these clinical factors are not reliable surrogate markers of anti-EGFR sensitivity, liquid biopsies are highly recommended for the selection of patients who are eligible for rechallenge (72). Completion of ongoing clinical trials will provide further evidence for the use of anti-EGFR-based therapy in this setting to extend the continuum of care in patients with mCRC.

## Author contributions

All authors contributed equally to the conception of the intellectual content and writing of the manuscript. All authors also reviewed any revisions that were made and provided their final approval of the manuscript.
